# The significance of component-resolved diagnostics in atopic dermatitis

**DOI:** 10.3389/fimmu.2026.1711854

**Published:** 2026-03-02

**Authors:** Małgorzata Błażowska, Julia Nowowiejska-Purpurowicz, Maria Tomasiak-Łozowska, Łukasz Błażowski, Iwona Flisiak

**Affiliations:** 1 Department of Dermatology and Venereology, Medical University of Bialystok, Bialystok, Poland; 2 Department of Allergology and Internal Medicine, Medical University of Bialystok, Bialystok, Poland; 3 Department of Allergology and Pulmonology, National Research Institute of Tuberculosis and Lung Diseases, Rabka-Zdroj, Poland; 4 Faculty of Medicine, Rzeszow University, Rzeszow, Poland

**Keywords:** allergen components, atopic dermatitis, atopic march, component resolved diagnostics, CRD, molecular allergy diagnostics, precision medicine

## Abstract

Atopic dermatitis (AD) is a common and chronic inflammatory skin disease characterized by an abnormal skin barrier, resulting in skin dryness, pruritus, and an increased risk of allergies and secondary infections. AD patients often show hypersensitivity to both food and airborne allergens. Component-resolved diagnostics (CRD) offers sIgE testing of individual allergen molecules and provides additional insights, especially in polysensitized patients, in case of sensitization to allergens with low abundance, low stability, or associated with risks of anaphylaxis. It enables the detection of genuine and cross-sensitization or the composition of an allergen immunotherapy vaccine. So far, the utility of CRD in AD has never been thoroughly analyzed. This review provides basic information about CRD and comprehensively summarizes its potential application in the personalized management of patients with AD. Molecular profiling of allergen components moves closer to explaining the mechanisms of development of different molecular endotypes and clinical phenotypes of AD and provides biomarkers of disease severity, autoimmune IgE responses, and therapeutic response, improving understanding of atopic dermatitis endotypes and treatment outcomes.

## 1 Introduction

Atopic dermatitis (AD) is a common, chronic inflammatory dermatosis accompanied by a persistent pruritus, often with a severe course, greatly influencing the patient’s life quality. The onset of the disease usually occurs in infants; however, it affects both children and adults, including elderly people (1–3).

The pathophysiology of AD is complex and involves genetic predisposition, skin barrier defect, abnormal immune response, and skin microbial disorders (1, 3–5). AD is related to abnormal type-2 immune response, often an elevated concentration of total immunoglobulin (Ig) E and IgE-mediated sensitization to various allergens, which, in some cases, plays a significant role in the exacerbation of AD as a triggering factor (2). Some patients present an IgE autoimmune response primarily induced by exogenous allergens that are homologous to the proteins of human epidermis (4).

The diagnosis of IgE-mediated sensitization is mainly based on skin prick tests (SPTs) using allergen extracts and specific IgE (sIgE) to allergen extracts (4, 6). However, the extract is a mixture of allergen components of various clinical relevance. Another restriction is that SPT may not be possible to perform in case of extensive and severe skin lesions. Component-resolved diagnostics (CRD) offers sIgE testing of individual allergen molecules and provides additional information, especially in polysensitized patients and in the case of allergens with low abundance, low stability, or associated with risks (7). This strategy is in line with the principles of precision medicine (6, 8). Molecular profiling of allergen components may initiate future research to recognize the environmental, genetic, and epigenetic factors responsible for the development of different clinical phenotypes and molecular endotypes of AD (4). However, so far, the use of such methods is not very widespread, being used more often by allergologists in allergy diagnostics in a broad sense than by dermatologists.

To date, data about the utility of CRD in the diagnosis and treatment of AD are limited (2). This review aims to summarize the current possibilities and applications of CRD in AD in clinical practice. The potential impact on the prediction of the course of the disease, risk of anaphylaxis, and treatment personalization or outcomes has been analyzed.

## 2 Methods

This is a narrative review of the current data available on the CRD application in AD. The manual search took place between 1 June 2025 and 15 January 2026. Online databases (PubMed, Scopus) were searched by two doctors (M.B., J.N-P.) using the following MeSH terms: “atopic dermatitis,” “component-resolved diagnostics,” “allergen components,” “CRD,” “molecular allergy diagnostics,” “MAD,” “precision allergy molecular diagnosis,” and “PAMD@.” We included papers that were published only in English. No publication date restrictions were set. We excluded papers that were duplicates and lacked sufficient methodological or conceptual detail. In case of disagreement about the inclusion of a particular article, a third doctor (M.T-Ł.) made the final decision.

## 3 Background of atopic dermatitis

AD is a common chronic inflammatory dermatosis with a relapsing, often severe course. The disease affects both children (13%) and adults (7%) and usually develops in infancy. Approximately 70% of patients may achieve remission before adolescence, whereas in 25%, AD continues into adulthood (1).

Clinical presentation includes eczema-like eruptions (erythema, papules, exudative lesions) of specific location depending on the age of the patient, excoriations, and dryness of the skin, resulting in pruritus. Long-term inflammatory process leads to lichenization (1).Managing patients with AD requires a wide range of treatments. Recommendations include trigger avoidance, application of a topical treatment (emollients, corticosteroids, calcineurin inhibitors, additional antimicrobials), allergen immunotherapy (AIT), systemic therapy (systemic corticosteroids, cyclosporine A, biologics, JAK inhibitors), and phototherapy (1).

## 4 A brief overview of the pathomechanism of atopic dermatitis

The pathophysiology of AD is complex and involves genetic predisposition, skin barrier defect, abnormal immune response, and skin microbial disorders. Impaired skin barrier, first of all, leads to increased water loss and insufficient skin moisture (1, 3, 5). Moreover, it promotes penetration of allergens and pathogens, which elicit immune sensitization and activation of the Th2-dependent response (type 2 inflammation) (1, 5). Initially, the inflammatory response involves epidermal antigen-presenting cells (APCs), such as Langerhans and dendritic cells (DCs). APCs bind the antigen and present it to Th2 cells (3, 5, 9, 10).

The major type 2 cytokines, IL-4 and IL-13, stimulate B lymphocytes to switch to allergen-specific immunoglobulin E (sIgE) production (1, 5, 8, 11–13). IgE antibodies can bind to two types of receptors: the high-affinity IgE receptor (FcϵRI) and the low-affinity receptor FcϵRII/CD23. FcϵRI is located on mast cells, basophils, Langerhans cells, and DCs; initiates Th2 response; and is responsible for allergic symptoms of type 1 hypersensitivity (5, 14–16). FcϵRII/CD23 is expressed by mast cells, monocytes, DCs, eosinophils, and platelets and plays a significant role in the regulation of IgE production and the moderation of IgE response (14–16).

Re-exposure to allergens elicits an IgE-dependent inflammatory process (10). Immunological response is associated with innate immunity cells such as DCs, innate lymphoid cells type 2 (ILC-2), mast cells, basophils, and eosinophils (1, 5, 17). IL-5, another Th2 cytokine, is responsible for eosinophil proliferation, differentiation, and susceptibility to allergic inflammatory reactions (12).

In summary, based on the current classification of hypersensitivity reactions, the pathophysiology of AD is driven by several mechanisms, such as immediate, cell-mediated (Th1, Th2, Th3), and epithelial-mediated hypersensitivity. Symptoms of AD may occur almost immediately after allergen exposure or may be delayed due to the development of type 2 inflammation, often triggered by epidermal barrier defect and alarmin activation (18). Moreover, some allergens, especially airborne, with a high degree of similarity with human epidermal proteins, stimulate the synthesis of allergen-specific IgE. As a result of cross-reactivity, IgE can induce type 2 inflammation after binding to homologous proteins in the epidermis (2). Sensitization does not always result in a clinical reaction, and it is essential to evaluate the actual risk of an allergic response through proper clinical assessment. Subclinical sensitization involves the production of IgE antibodies without the presence of noticeable allergic symptoms (19). Notably, AD may be associated with other conditions of similar pathogenesis (5, 10).

## 5 Atopic march

The occurrence of allergic diseases is significantly higher in patients with AD than in the general population (9). The development of other atopic disorders, such as food allergy (FA), allergic asthma (AA), and allergic rhinitis (AR) in patients suffering from AD, is called atopic march (5, 10). This refers to the natural course of allergy and is mainly associated with the early onset of AD (5, 19, 20).

Approximately 30% of children with AD suffer from FA (9) and *vice versa*—persistent IgE-mediated FA is associated with early-onset or severe AD, as well as with AR and AA (5, 19). The likelihood of developing AR and AA is approximately 50%–75% of infants and young children with AD, and the prevalence of AA concerns approximately 20% and increases to more than 60% in children with mild and severe AD, respectively (5).

Patients with AD are often polysensitized and demonstrate high concentrations of total IgE (tIgE) and sIgE (3, 4, 10, 21), particularly toward food, airborne, and microbial allergens, such as *Malassezia sympodialis* (7, 17, 21, 22). Moreover, patients with AD often develop IgE-mediated sensitization to self-antigens (2, 21).

In clinical practice, relevant symptoms reported by patients have to be taken into account to further guide the diagnostic process. All recommended allergic tests have to be carefully chosen and interpreted with caution (23).

## 6 Component-resolved diagnostics

**Figure 1:**
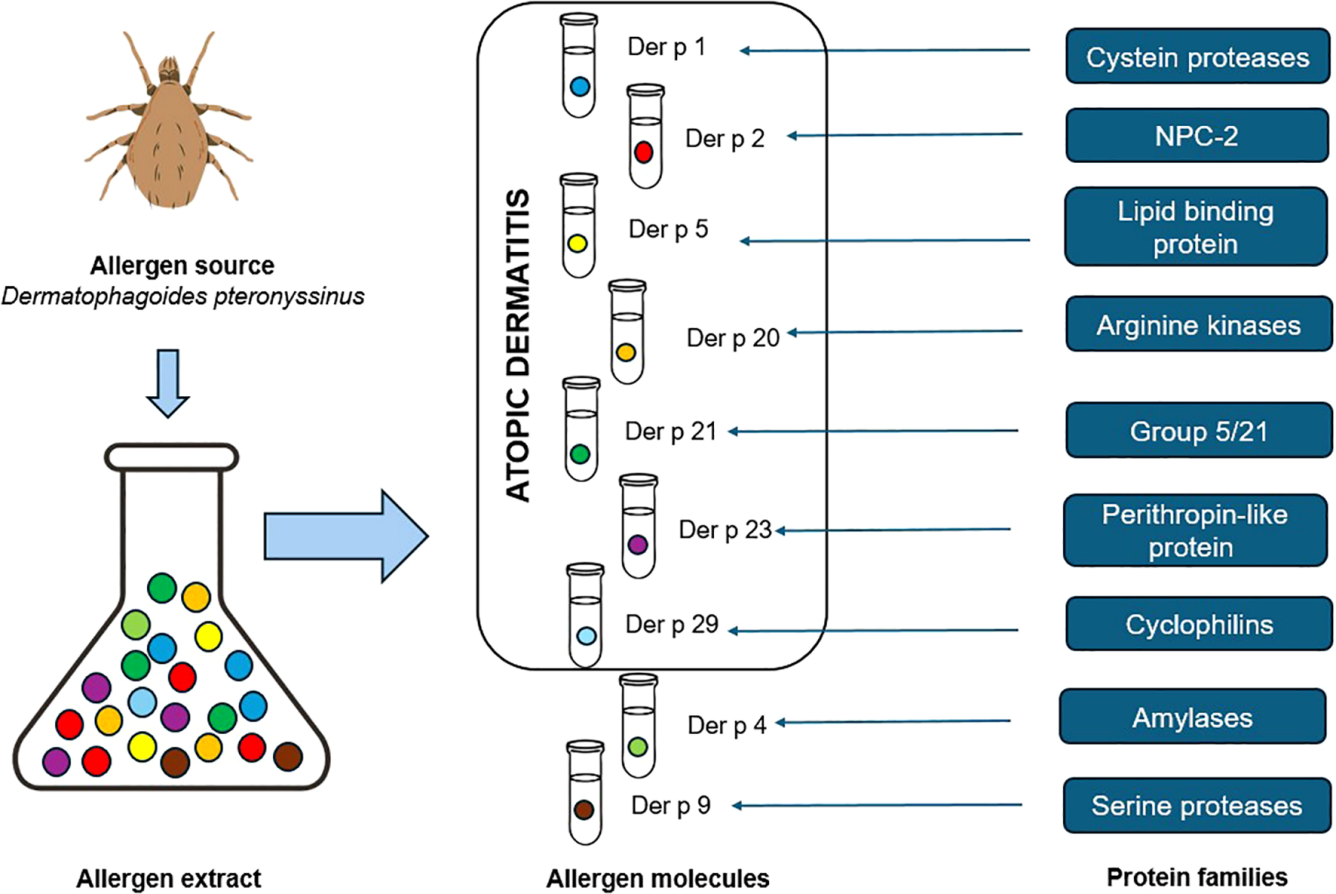
Allergen source, allergen extract, allergen molecules, and allergen families according to the CRD principles (7, 24, 25).

Conventional common allergic tests, such as SPTs and serum sIgE tests, are based on allergen extracts derived from natural sources. However, the allergen extract is a mixture of allergen components (molecular allergens) and non-allergenic proteins (**Figure 1**) (17, 24, 25). In a molecular sense, only the binding of allergen-specific IgE with the epitope of the allergen molecule initiates the allergic immune response (7, 11, 25, 26).

CRD establishes the patient’s IgE reactivity profile at a molecular allergen level and moves allergology into the era of precision medicine. CRD enables the identification of genuine and cross-reactive allergens as well as anaphylaxis risk assessment, the prescription of allergen immunotherapy, and the prediction of the natural course of allergic disease (6, 17, 24, 27).

Allergen molecules are classified as major or minor allergens. Major allergens induce IgE sensitization in at least 50% of allergic patients in relation to allergen source. In comparison, minor allergens are responsible for inducing IgE response in less than 50% of patients (7, 8, 25, 26). Allergens demonstrating similarity form the specific allergen families, and their homology is often the underlying cause of cross-reactions (8, 25, 26). CRD eliminates the problem of both false-positive results owing to cross-sensitization and false-negative results due to the absence of allergen molecules in the allergen extract (11, 24).

Molecular allergens are derived from natural sources or may be produced in recombinant form. Since allergen extracts may contain varying amounts of allergen molecules, which can be deficient, a precise definition of personal IgE reactivity profiles is achieved by using mostly recombinant allergens (7, 8, 11, 24, 27).

Furthermore, recombinant allergens do not contain cross-reacting carbohydrate determinants (CCDs), which are typically found in natural plant allergens but not in humans (however, this varies according to the expression system used, since some systems can add CCDs to the recombinant protein) (28). CCDs are not clinically relevant but can induce the synthesis of CCD-specific sIgE antibodies, which give false-positive IgE results to extracts or natural allergens (27).

CRD is performed *in vitro* with single or multiplex diagnostic tests. The singleplex assays are characterized by a high sensitivity, while the multiplex tests give a broad sensitization profile, especially in polysensitized patients, but have a potentially lower sensitivity (17, 24, 27). Notably, a positive sensitization to allergen extracts and molecules represents risk for allergic disease and is clinically relevant if they are accompanied by corresponding symptoms (7).

## 7 The role of allergen molecules in IgE-mediated inflammation and induction of flares in atopic dermatitis

**Figure 2:**
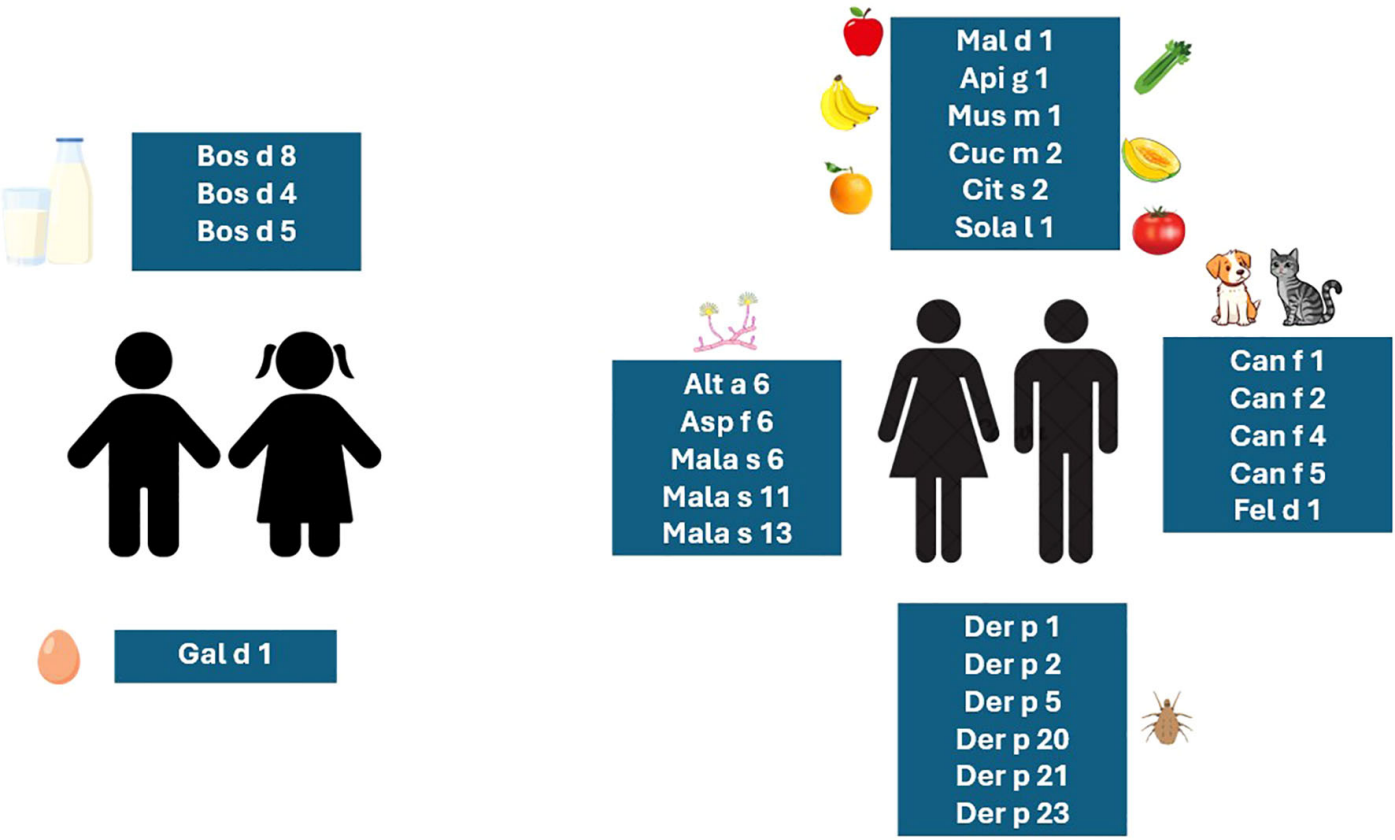
The relevant molecular allergens responsible for exacerbations of AD in children and adults (2, 7, 9, 11, 13, 17, 21).

Allergen exposure may cause an exacerbation of AD in sensitized patients (29). Food allergens such as egg and cow’s milk (CM) are mainly responsible for inducing flares of AD in infants and small children, whereas pollen-related cross-reactive food proteins and airborne allergens such as house dust mites (HDMs), molds, animal dander, or pollens are more often a cause of exacerbation in older children, teenagers, and adults (**Figure 2**) (9, 13, 22). In one study, it has been shown that in 3-month-old children with AD, FA is six times more frequent compared with healthy controls (9). Among food allergens, the severity of AD is related to a major egg allergen, Gal d 1 (ovomucoid), and major CM allergens such as Bos d 8 (casein) and whey proteins Bos d 4 (α-lactalbumin) and Bos d 5 (β-lactoglobulin). Of the cross-reactive, birch pollen-related food allergens, from the pathogenesis-related 10 subfamily (PR-10) such as Mal d 1 (apple), Pru p 1 (peach), Gly m 4 (soy), and Api g 1 (celery), as well as grass pollen-related food allergens from the profilin family such as Mus m 1 (banana), Cuc m 2 (melon), Cit s 2 (orange), and Sola l 1 (tomato), are also involved in AD exacerbations (7, 21). On the other hand, being sensitized to even a single inhalant allergen can significantly increase the risk of developing AD (11, 13).

AD severity and persistent eczematic lesions are associated with major dog allergens such as Can f 1, Can f 2, Can f 4, Can f 5, and Can f 6; major cat allergen Fel d 1; storage mite allergens Gly d 2 and Lep d 2; and HDM allergens Der p 1, Der p 2, Der p 20, Der p 21, and Der p 23 (11, 17). Many studies point out that HDM sensitization is considered a pivotal factor starting the atopic march and highlight it as the most relevant allergens in the induction of flares in patients suffering from AD (17, 29, 30).

For example, the major HDM allergen, Der p 1, facilitates microbial superinfection as well as sensitization to other allergens due to its enzymatic protease activity and disruption of tight junctions of keratinocytes. Hence, HDM allergens play a crucial role in initiating type 2 response through epidermal barrier damage and the release of alarmins (17, 29). Furthermore, sensitization to a particular HDM allergen may influence the clinical picture of AD. Moreover, more than 75% patients with severe AD have a high concentration of sIgE against Der p 20 (30).

In addition, sensitization to mold (Asp f 6, Cla h 8) and yeast (Mala s 6, Mala s 11) allergens also correlates with the severity of AD. Especially, a high concentration of sIgE toward Asp f 6, manganese superoxide dismutase (MnSOD) from *Aspergillus fumigatus*, and toward Mala s 11, MnSOD from *M. sympodialis*, is characteristic of severe AD with an autoimmune phenotype (2, 31). Moreover, eczematous lesions covering the neck and head are related to the presence of sIgE against *M. sympodialis* antigens, thus can be described and used as a marker for the severity of AD (7).

As mentioned above, patients with AD are often polysensitized, and those with co-existing FA may have a significant risk of food-induced anaphylaxis. In such subjects, CRD is especially helpful.

## 8 The role of component-resolved diagnostics in the assessment of risk of food anaphylaxis in patients with polysensitization and atopic dermatitis

Anaphylaxis is a sudden, promptly evolving life-threatening systemic allergic reaction (32, 33), mainly mediated by an IgE-dependent response and commonly induced by food, venom, or drugs (34). The risk of severe reactions, such as fatal or almost fatal anaphylaxis, mainly concerns adolescents and young adults with FA (19). CRD can help identify the specific allergen molecule responsible for an immediate allergic reaction and provide additional insights into the likelihood of anaphylaxis. In some cases, it can be the only method enabling the diagnosis of the causal factor (35). CRD is also crucial in cases where the cause of anaphylaxis is unknown, and the event is classified as idiopathic (35).

Various allergen molecules may be associated with different severities of allergic reactions. In case of FA, sensitization to storage proteins from peanuts, nuts, and seeds increases the risk of anaphylaxis (36). This applies in particular to 2S albumins from peanut (Ara h 2), cashew (Ana o 3), and hazelnut (Cor a 14), as well as lipid transfer proteins (LPTs), such as Pru p 3 from peach, tropomyosin from shrimp (Pen a 1), parvalbumin from fish (Gad m 1), casein from milk (Bos d 8), and ovomucoid (Gal d 1) from hen’s egg (7, 19, 26, 35). Therefore, CRD may be a worthwhile diagnostic and preventive tool (26).

## 9 The role of component-resolved diagnostics in the prediction of the natural history of atopic dermatitis, its progression, prognosis, and treatment

Early-onset AD is often associated with high concentrations of tIgE and is considered a risk factor for other atopic diseases such as FA, asthma, AR, and pollen-food allergy syndrome (PFAS) (3, 19, 22, 37). Early exposure to food allergens through inflamed and damaged skin can lead to the development of type 2 immune response and IgE-mediated FA (4, 19). On a vast group of 2,184 children, it has been proven that high sIgE toward CM, hen’s egg, and peanut was significant in infants who developed AD before the end of the third month of life. For comparison, when AD manifested after the first year of life, the risk decreased (22) and late-onset AD does not seem to be associated with food sensitization (37). Additionally, the earlier the AD lesions occur and the more severe they are, the more commonly extremely sIgE sensitizations against food can be noted (22). Moreover, colonization of methicillin-resistant *Staphylococcus aureus* (MRSA) contributes to a higher risk of FA (9). As a result, one-third of children with AD develop IgE-FA (19). Sensitization to the yeast *M. sympodialis* may play a role in developing the head-neck-shoulder phenotype of AD, with the incidence of sIgE in up to 67% of patients (22).

So far, there has been only one original study assessing the potential utility of CRD in AD patients. The authors tested over 100 components of 45 allergens (food, airborne, venom) and concluded that CRD results corresponded to SPT. However, the main limitation was a very low number of participants (25), and the methodology was slightly different in comparison with contemporary tests (38).

Several studies investigated the association between hypersensitivity to specific molecular components and the occurrence of other atopic diseases, with only a few assessing AD in particular. Considering the relationship between AD, AR, and asthma, the study by Vaňková et al. on a group of 104 AD patients found that the prevalence of AR and asthma was comparable to previously reported data, as mentioned earlier (5, 17). This study has proven that skin barrier disruption can trigger type 2 response and suggests a contribution to progression from AD to AR and asthma. The risk of developing asthma or rhinitis in AD patients might especially increase IgE sensitization to HDM and animal dander (22, 39).

The high risk of developing IgE sensitization to major HDM allergen, Der p 23, in a group of 191 German children with a history of AD was observed in the study of Fochert et al. (40) They stated that sensitization to Der p 23 correlates with developing asthma later on (40). Furthermore, sensitization to other molecular components of HDM, such as Der p 1, Der p 2, and Der p 5, is related to asthma (17, 30, 33).

Walsemann et al. confirmed the relationship between sensitization to more than three mite allergens and the occurrence of AD and/or allergic asthma. AD patients were more often sensitized to Der p 5, Der p 20, and Der p 21, among others. In subjects with AD, sensitization to Der p 5, Der p 10, Der p 13, Der p 20, and Der p 21 was related to a severe course of AD, unlike moderate and mild cases. The sIgE concentration for Der p 20 above 80 kU/L was associated with severe AD in 75% of patients (30). To conclude, sIgE against Der p 20 appears to be a promising biomarker of the severity of AD (30).

One of the studies reported that the major allergens of HDM—Der p 1, Der p 2, and Der p 23—were present in more than 86% of European subjects with AD highly exposed to HDM. Der p 23 was highlighted as a major *Dermatophagoides pteronyssinus* allergen in severe AD and was identified in 97% individuals, followed by Der p 2 in 95% and Der p 1 in 86.25% (41). Der p 5, Der p 7, and Der p 21 showed mid-tier seroprevalence (41).

In the treatment of AD patients sensitized to Der p 1, Der p 2, and/or Der p 23, specific AIT may be useful (11, 42). According to the study of Bogacz-Piaseczynska et al., AIT toward HDM can provide complementary treatment in patients with moderate to severe AD and sensitized to HDM (43).

The majority of reviewed studies focused on HDM molecular components. Less is known about the impact of other molecular allergens. The main limitation of the extracted research is certainly a relatively low number of enrolled participants and the diverse methodology.

Engeroff et al. pointed out that the low-affinity receptor for IgE, FcϵRII/CD23, expressed on B cells, contributes to the regulation of immune responses through IgE-immunocomplexes (IgE-ICs), unlike type I hypersensitivity, which is mediated by allergen-specific IgE binding to FcϵRI on mast cells and basophils. IgE cannot bind simultaneously to both FcϵRI and FcϵRII/CD23 (14). Free IgE elicits a perceptible inflammatory allergic response accompanied by allergic effector cells, while IgE-IC maintains a non-inflammatory response without degranulation of mast cells and basophils. Based on Engeroff’s study, Celakovska et al. focused on the interaction between the expression of the CD23 molecule on B lymphocytes and the level of sIgE against molecular components of pollen, Niemann-Pick protein type C2 family (NPC2), lipocalins, uteroglobins, molds, and yeast in AD patients (15, 16). In those treated with dupilumab, the most widely used biological agent in AD therapy, a correlation between higher CD23 expression on B cells and sIgE against Feld 1 (cat), Bla g 9 (German cockroach), and Cla h 8 (*Cladosporium herbarum*) was confirmed compared to patients untreated with dupilumab (16). Moreover, patients without dupilumab therapy revealed increased free sIgE to storage mite allergens from the NPC2 family (Gly d 2, Lep d 2), dog lipocalins (Can f 1, Can f 2), and arginine kinase from German cockroach (Bla g 9). The findings suggest that dupilumab improves CD23-mediated regulation by diminishing excessive free IgE, thus reducing allergic inflammation (15, 28).

## 10 The autoimmune IgE response in atopic dermatitis

The role of the autoimmune IgE response in AD is currently an intensely discussed issue (43). The latest, multicenter review confirmed the role of IgE-mediated autoimmunity in the pathophysiology of AD and other chronic inflammatory diseases (CIDs), distinguishing it from allergy (43). The research of Scala et al. focused on IgE responses to 48 human-homologous exogenous molecular allergens (HEMAs) using microarray tests (2). They observed that IgE antibodies to the majority of HEMAs occur more frequently in patients suffering from severe AD compared to others. It was closely linked to certain skin conditions like flexural dermatitis, head and neck dermatitis, or widespread eczema. Based on those results, the IgE molecular-mimicry index (IgE-MMI) was described (2).

IgE-MMI determined the frequency of IgE antibody against one or more of the five HEMA protein families (**Table 1**), which consist of arginine kinase (AK) from HDM and cockroach (Der p 20 and Bla g 9), enolase (ENO) from *Alternaria alternata* (Alt a 6), cyclophilin (CYP) from *M. sympodialis* (Mala s 6), lipocalin from cat and dog (Fel d 7 and Can f 1), and MnSOD from *M. sympodialis* and *A. fumigatus* (Mala s 11, Asp f 6) (2). Another *M. sympodialis* allergen, Mala s 13 (thioredoxin), has a 45% sequence identity with human thioredoxins and can also lead to cross-reactivity and autoimmune IgE response in individuals with AD (7).

**Table 1:** Protein families involved in the IgE molecular-mimicry index (MMI) and cross-reactive airborne allergens from these families (2, 7, 23).

Protein family	Cross-reactive airborne allergens	Allergen source
Manganese superoxide dismutases (MnSOD)	Alt a 14 Asp f 6 Hev b 10 Mala s 11	*Alternaria alternata* *Aspergillus fumigatus* Latex *Malassezia sympodialis*
Cyclophilins (CYP)	Asp f 11 Asp f 27 Der p 29 Mala s 6 Per a 18	*Aspergillus fumigatus* *Aspergillus fumigatus* *Dermatophagoides pteronyssinus*, house dust mite *Malassezia sympodialis* *Periplaneta americana*, American cockroach
Enolases (ENO)	Alt a 6 Asp f 22 Cla h 6 Hev b 9 Per a 14	*Alternaria alternata* *Aspergillus fumigatus* *Cladosporium herbarum* Latex *Periplaneta americana*, American cockroach
Lipocalins	Can f 1 Fel d 7	Dog Cat
Arginine kinases (AK)	Bla g 9 Der p 20 Per a 9 Tyr p 20	*Blatella germanica*, German cockroach *Dermatophagoides pteronyssinus*, house dust mite *Periplaneta americana*, American cockroach *Tyrophagus putrescentiae*, storage mite

According to Scala et al., subjects with a severe course of AD demonstrated increased IgE responses to many HEMAs compared to patients in remission. Thus, IgE to HEMAs and especially positive IgE-MMI are considered valuable biomarkers of AD severity. Moreover, the presence of IgE antibodies against enolase (ENO) was correlated with the poor response to dupilumab, in contrast to the presence of IgE against MnSOD and NPC-2 (2).

## 11 Conclusions

Skin barrier disruption is the main culprit in inducing sensitization and allergy in patients with atopic dermatitis. Maintaining a proper skin barrier and early treatment may prevent not only the development of food allergy but also other atopic diseases such as asthma and allergic rhinitis.

**Figure 3:**
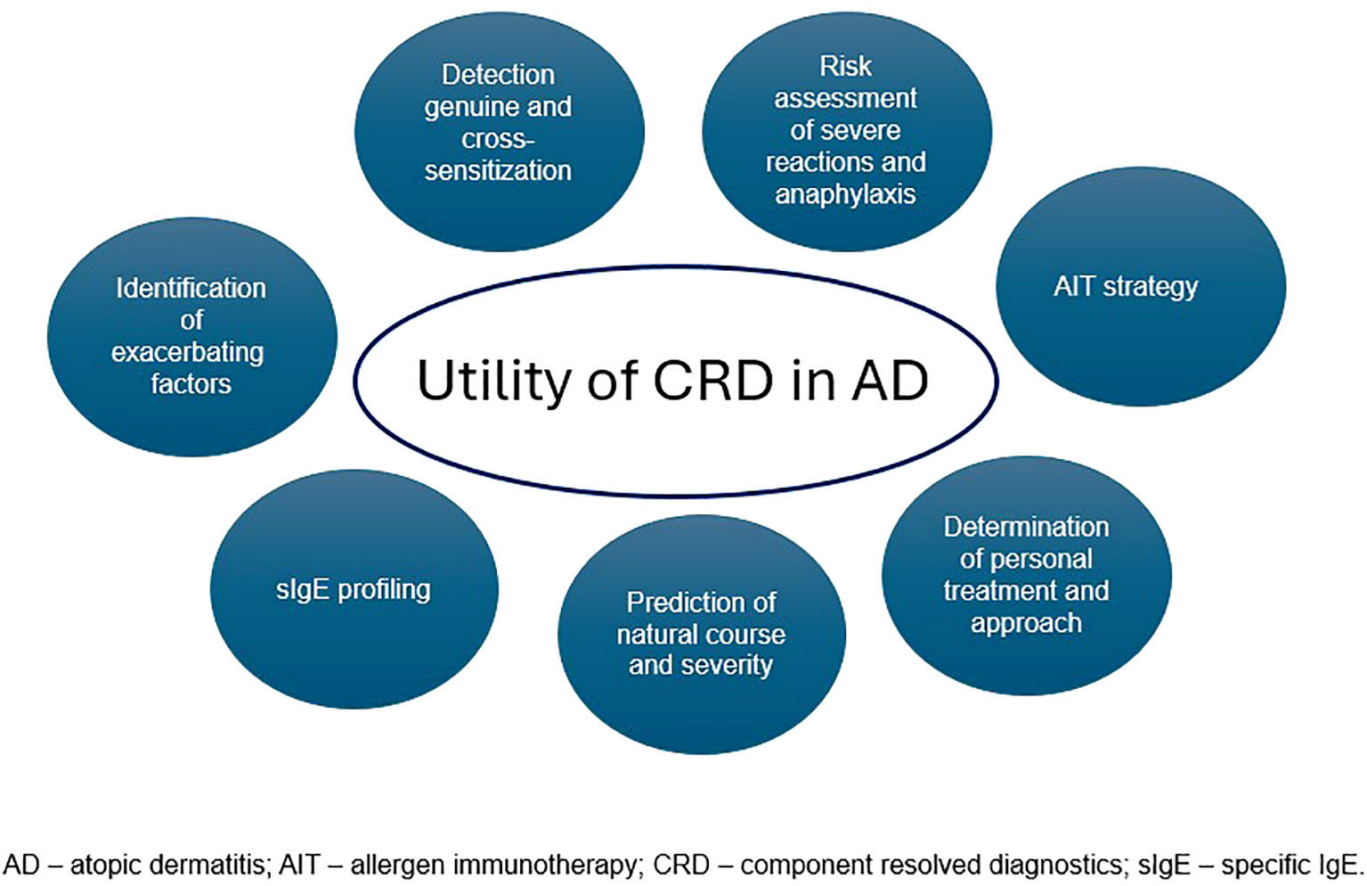
Practical application of CRD in patients with AD.

Component-resolved diagnostics establishes the patient’s sensitization profile at a molecular level and moves allergology into the era of precision medicine. It enables the identification of genuine or cross-sensitization as well as anaphylaxis risk assessment, prediction of natural course, severity of atopic dermatitis, and progression toward other atopic diseases (**Figure 3**). However, this is a novel method that is gaining interest and importance in everyday clinical practice.

Regarding infants and little children, mainly food allergens such as egg and cow’s milk are responsible for inducing flares, whereas pollen-related foods and especially inhalant allergens are more often a cause of exacerbation of atopic dermatitis in older children, teenagers, and adults. Being sensitized to even a single inhalant allergen can greatly increase the risk of developing atopic dermatitis, and house dust mite allergens are the most relevant in the induction of flares in these patients. IgE sensitization to Der p 20 seems an up-and-coming biomarker. Allergen immunotherapy toward house dust mite can provide complementary treatment in sensitized patients with moderate-to-severe atopic dermatitis.

In addition, sensitization to human-homologous exogenous molecular allergens and a positive molecular-mimicry index are considered valuable biomarkers of the severity of atopic dermatitis, autoimmune IgE-mediated response, and treatment response to dupilumab.

Molecular profiling of allergen components moves closer to explaining the mechanisms of development of different clinical phenotypes and molecular endotypes of atopic dermatitis, as well as response to treatment. Future studies and clinical observations will certainly determine more clearly the utility of CRD in atopic dermatitis patients.
